# MicroRNA-181b Serves as a Circulating Biomarker and Regulates Inflammation in Heart Failure

**DOI:** 10.1155/2021/4572282

**Published:** 2021-07-01

**Authors:** Hongxiao Yang, Lina Shan, Yunan Gao, Lin Li, Guifen Xu, Bin Wang, Xiaoxue Yin, Chengfang Gao, Jiaren Liu, Wei Yang

**Affiliations:** ^1^Department of Cardiology, The Fourth Affiliated Hospital of Harbin Medical University, 37 Yiyuan Street Harbin, Heilongjiang, China 150001; ^2^Department of Cardiology, The First Affiliated Hospital of Harbin Medical University, 23 Youzheng Street, Harbin, Heilongjiang, China 150001; ^3^Department of Clinical Laboratory, The Fourth Affiliated Hospital of Harbin Medical University, 37 Yiyuan Street, Harbin, Heilongjiang, China 150001.

## Abstract

Heart failure (HF) is the typical terminal stage of cardiac diseases involving inflammatory states. The function of microRNAs (miRNAs) in the progress of HF remains poorly understood. In this study, real-time PCR results showed a decreased expression of miRNA-181b (miR-181b) in HF patients compared with healthy individuals. Besides, miR-181b expressions were negatively correlated with hypersensitive C-reactive protein (hsCRP) levels in the serum of HF patients. Receiver operator characteristic (ROC) curve analysis showed that miR-181b was a diagnostic predictor of HF, and the area under the curve was 0.970 (DCM-induced HF group) and 0.962 (ICM-induced HF group). Strikingly, in HF rats induced by isoproterenol (ISO), the expression of miR-181b of heart tissue was suppressed before tumor necrosis factor-alpha (TNF-*α*), interleukin-1*β* (IL-1*β*), and interleukin-6 (IL-6) increase, as revealed by western blot and real-time PCR. Besides, the overexpression of miR-181b also decreased the expression of TNF-*α*, IL-1*β*, and IL-6 in lipopolysaccharide- (LPS-) induced neonatal cardiomyocytes. In conclusion, our results revealed that miR-181b might be a potential biomarker for HF and provided a novel target for anti-inflammatory therapy.

## 1. Introduction

Heart failure (HF), as the typical terminal stage of cardiovascular diseases, has high morbidity and mortality worldwide. Myocardium ischemia and dilated cardiomyopathy are two important pathological processes leading to the progressing of HF, and both of which are involved in inflammatory mechanisms [1]. In HF, proinflammatory cytokines such as IL-6 and TNF-*α* are considered to contribute to disease progression by exerting on the heart and circulation [2].

Several studies have shown that HF patients are characterized by sustained immune activation and elevated levels of circulating proinflammatory cytokines, such as TNF-*α*, IL-6, and IL-1*β* [3]. Proinflammatory signaling is mediated by various inflammatory mediators, which can cause apoptosis, fibrosis, and ultimately lead to adverse ventricular remodeling [4].

Biomarkers play an important role in the clinical management and risk stratification for cardiovascular diseases (CVD) and HF [5]. Blood is the most common diagnostic biosample for clinical testing; others include a tissue and urine [6]. In addition, recent trends indicate that the measurement of diagnostic biomarkers in novel biological specimens (such as the saliva) is becoming increasingly popular due to its noninvasive nature [7–9]. In routine clinical analysis, protein assays are the most widely used, followed by cellular and molecular assays; however, the clinical application of microRNA analysis has recently received a lot of attention [6].

MicroRNAs (miRNAs) are involved in diverse physiologic and pathologic processes, including cellular proliferation, differentiation, angiogenesis, and apoptosis. Importantly, abundant and stable miRNAs can be detected in plasma, and their expression is frequently dysregulated in diseases [10]. Multiple circulation miRNAs have been reported as an ideal cluster of blood-based biomarkers for many diseases such as cancer and cardiac diseases [11, 12]. However, the mechanisms underlying the miRNA interaction with the inflammation in HF have not been elucidated.

The miR-181 family comprises four miRNAs: miR-181a, miR-181b, miR-181c, and miR-181d. In most of the studies previously, miR-181b is reported as a kind of carcinogenic gene. Interestingly, miR-181b is also related to vascular inflammation [13]. This fact aroused our hypothesis about the identification of miR-181b as the regulator of the inflammatory process in HF.

The involvement of differential miRNA expression in inflammation during the development of HF is far from fully elucidated. Based on these previous studies, the present study is aimed at investigating the changes of serum miR-181b expressions and its correlation with serum inflammatory cytokines in patients with HF and further studying its regulatory effect on the expression of inflammatory cytokines in HF, to find new biomarkers and intervention targets for clinical treatment of HF.

## 2. Methods

### 2.1. Definition of DCM/ICM-Induced HF

We conducted a clinical study analysis of healthy individuals and HF patients induced by DCM or ICM. In Table 1, the patients of HF presented the following inclusion criteria: HF was diagnosed according to the recent guidelines [14] with reduced left ventricular systolic function (LVEF < 50%) and increased NT-proBNP (>300 pg/mL). Patients with DCM were eligible to enroll after their diagnosis was confirmed, as defined by left ventricular or biventricular systolic dysfunction and dilatation that could not be explained by abnormal load conditions or coronary artery disease [15]. Patients with ICM were diagnosed with a history of myocardial infarction or revascularization, or a stenosis of 75% or more of the left main or proximal left anterior descending coronary artery, or stenosis of two or more epicardial vessels [16]. Two-dimensional and M-mode echocardiography was used to determine the LVEF and LVEDD. Healthy individuals presented no systemic diseases, overt cardiac disorders, or abnormal inflammatory indexes such as hsCRP and did not take any drugs.

Exclusion criteria were meeting one or more of the following: (1) age < 18; (2) patients with systemic complications such as malignant tumor, autoimmune disease, endocrine disease, or end-stage renal insufficiency; (3) blood-borne infectious diseases, including human immunodeficiency virus/acquired immunodeficiency syndrome, hepatitis B, and hepatitis C; (4) known hypertrophic/alcoholic/chemical/peripartum cardiomyopathy; (5) infection; and (6) pulmonary disease.

The trial protocol conformed to the Declaration of Helsinki's ethical guidelines and was approved by the Ethics Committees of the First Affiliated Hospital of Harbin Medical University. All subjects provided written informed consent.

### 2.2. Animals

Healthy female Wistar rats weighing 200-220 g were used in the present study and housed in a standard animal room with free eating and drinking. All experimental procedures were performed following the Guide for the Care and Use of Laboratory Animals of the First Affiliated Hospital of Harbin Medical University. We took the utmost care to minimize suffering on the part of the rats. Rats were sacrificed by cervical dislocation for analysis.

### 2.3. HF Models and Echocardiographic Measurement

The HF rat model was achieved by intraperitoneal injection of isoproterenol (ISO). Each rat was daily administered 1 mg/kg body weight ISO or saline vehicle for one month or three months.

One month and three months after injection of ISO or saline vehicle, echocardiography (Sonos7500 Phillips) was performed to test cardiac function. The measurement was performed under isoflurane anesthesia to alleviate any pain and suffering on the rats' part.

### 2.4. Primary Culture of Rat Neonatal Cardiomyocytes

As mentioned above, Wistar rats aged 1 to 3 days were sacrificed by cervical dislocation [17]. The neonatal heart is chopped up and placed in 0.25% trypsin. The mixed cell suspensions were centrifuged and resuspended in Dulbecco's modified Eagle's medium (DMEM) supplemented with 10% fetal bovine serum, 100 U/mL penicillin, and 100 *μ*g/mL streptomycin. We place the resuspension on the culture dish for 90 minutes to make the fibroblasts preferentially attach to the culture dish's bottom. Then, we removed the media containing cardiomyocytes to the new culture dishes. The cardiomyocytes were incubated in a humid atmosphere of 37°C, 5% CO_2_, and 95% air.

### 2.5. miRNA Transfection

According to the manufacturer's instructions, transfection was accomplished with Lipofectamine 2000 transfection reagent from Invitrogen. miR-181b mimics or miRNA negative control was transfected at 20 nM concentration, and miRNA-181b inhibitor or miRNA inhibitor negative control was transfected at 50 nM concentration. Cardiomyocytes were allowed to grow for 24 hours before lipopolysaccharide (LPS) treatment. Cardiomyocytes were used for real-time PCR and western blot.

### 2.6. RNA Isolation and Real-Time PCR

All participants were told to avoid strenuous exercise and alcohol consumption within 24 h before blood collection. Peripheral blood samples were drawn from cubital vein in fasting state before 9 a.m. According to the manufacturer's protocol, human serum's total RNA was isolated with TRIzol LS (Ambion 10296-028). Meanwhile, total RNA was isolated from the rat model heart tissue and neonatal rat cardiomyocytes using TRIzol. RNA content and primary were assessed by NanoDrop™ 2000c spectrophotometer (Thermo Scientific). Reverse transcription was with a reverse transcription kit (Promega Cat. #A3500) (Table 2). According to the manufacturer's protocol, real-time PCR analysis was performed using the ABI 7500 Real-Time PCR system (Table 2).

### 2.7. Western Blot

Lysis buffer was used to extract total protein from neonatal cardiomyocytes or heart tissue for western blot. The protein sample (80 *μ*g) was separated by SDS-PAGE and blotted on a nitrocellulose membrane. The blots were blocked with 5% skim milk at room temperature for 1 hour, then detected with primary antibody including TNF-*α* (1 : 200 dilution, no. MAB510, R&D), IL-*β* (1 : 200 dilution, no. MAB5061, R&D), IL-6 (1 : 500 dilution, no. MAB5011, R&D), and GAPDH (1 : 1000 dilution, no. TA-08, ZSGB). After incubation overnight at 4°C, the membrane was rinsed with PBS-T and incubated with the secondary antibody at room temperature for 1 hour. Finally, we collected the western blot bands through the imaging system. GAPDH was used as the control for equal loading of the protein.

### 2.8. Statistical Analysis

Continuous variables were presented as means ± standard error of the mean (SEM), and categorical variables were represented as counts (percentages). Baseline characteristics were compared using chi-square test for categorical variables, and analysis of variance (one-way ANOVA) was used for multiple comparisons, while an unpaired *t*-test was used for comparisons between two groups. Linear regression models were employed to examine associations of serum miR-181b level and hsCRP level. Receiver operator characteristic (ROC) curves, which cross different cut-off levels of logistic regression model prediction probability, were used to distinguish HF cases from healthy controls. The area under the ROC curve (AUC) was used to evaluate the diagnostic accuracy of miRNAs. All data analyses were performed using the SPSS for Windows version 17.0, and *P* < 0.05 was considered to be statistically significant.

## 3. Result

### 3.1. Circulating miR-181b Levels Are Significantly Reduced in Patients with DCM/ICM-Induced HF

**Figure 1:**
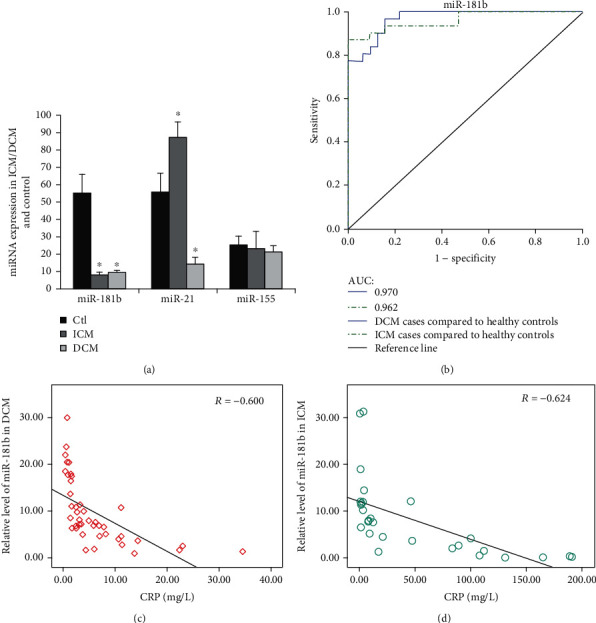
Expressions of miR-181b were downregulated in serum from human subjects with HF and correlated with established biomarkers of HF. (a) mMiR-181b, miR-21, and miR-155 expressions were detected by qPCR in serum from ICM-induced HF patients (*n* = 31), DCM-induced HF patients (*n* = 41), and healthy individuals (*n* = 31). Data are shown as mean ± SEM. ^∗^*P* < 0.05 compared with the control group. (b) Diagnostic performance of the relative abundances of miR-181b. AUC-ROC differentiates DCM-induced HF patients from normal subjects (miR-181b, 0.970) and differentiates ICM-induced HF patients from normal subjects (miR-181b, 0.962). (c, d) Correlation between miR-181b level and hsCRP level in the serum samples of HF patients. Pearson's correlation coefficients: *R* = −0.600 (*P* < 0.001, DCM group) and *R* = −0.624 (*P* < 0.001, ICM group).

To identify whether miR-181b is involved in the process of HF, we compared miR-181b expressions in serum in DCM/ICM-induced HF patients and healthy individuals. Serum miR181b expressions were reduced in 41 DCM- and 31 ICM-induced HF patients (by about 46% and 45%, respectively, comparing with healthy individuals) (Figure 1(a) and Table 1). Furthermore, we investigated whether HF influenced the expression of additional miRNAs. We determined the expressions of miR-21 and miR-155 in the serum in DCM-induced HF patients, ICM-induced HF patients, and healthy individuals. The miR-21 expression showed a decrease of approximately 41% in the DCM group, whereas we found it was upregulated 27% in the ICM group. In contrast, the miR-155 expression showed no notable differences between the three study groups (Figure 1(a)). The array data were confirmed by real-time PCR analysis. Regarding the effect of HF alone, expressions of miR-181b were significantly reduced in the DCM and ICM HF patients compared with healthy individuals, whereas miR-21 expressions were dysregulated.

### 3.2. The Downregulation of miR-181b Is Related to the Severity of the Disease in Patients with HF and the Diagnostic Performance of miR-181b

To investigate whether miR-181b is correlated with the established biomarkers of cardiac injury/cardiac function, we compared the expressions of miR-181b, NT-proBNP, and hsCRP in the serum of HF patients caused by DCM/ICM and healthy individuals. In patients with HF caused by DCM and ICM, miR-181b was negatively correlated with hsCRP (Figure 1(c), correlation coefficient: -0.600, *P* < 0.001 and Figure 1(d), correlation coefficient:-0.624, *P* < 0.001). Consistently, miR-181b was not related to NT-proBNP. Besides, miR-181b distinguished DCM- and ICM-induced HF patients from healthy controls with the area under the curve (AUC) of 0.970 (95% confidence interval, 0.937 to 1.000) and 0.962 (95% confidence interval, 0.917 to 1.000), separately (Figure 1(b)). These data suggest that miR-181b might be a potential novel biomarker for HF associated with ICM/DCM. However, these findings still need replication and validation in larger cohorts of CHF patients.

### 3.3. The Expression of miR-181b Is Downregulated in the Early Stage of HF before the Expression of TNF-*α*, IL-1*β*, and IL-6 Increase In Vivo

**Figure 2:**
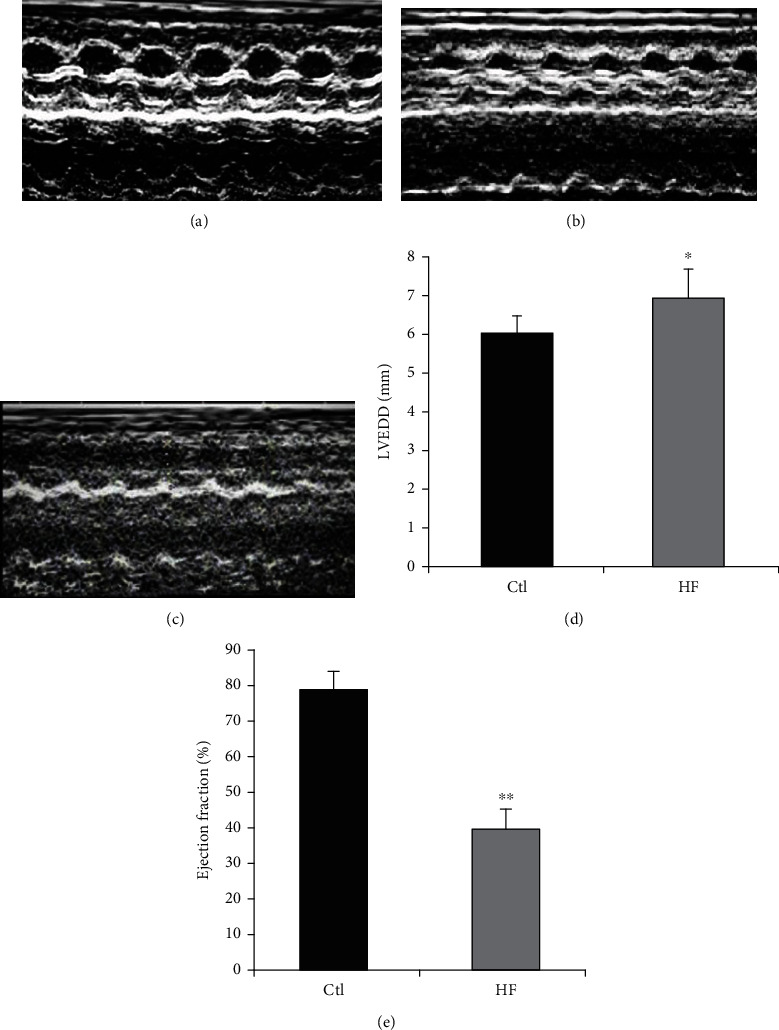
Echocardiography. (a–c) 2D M-mode and Doppler echocardiography were performed on rat intraperitoneal injection of saline vehicle, intraperitoneal injection of ISO for one month, and intraperitoneal injection of ISO for three months. (d) Left ventricular end-diastolic dimension (LVEDD) of rats with intraperitoneal injection of ISO for three months. (e) Ejection fraction (EF) of rats with intraperitoneal injection of ISO for three months. Data are expressed as mean ± SEM, *n* = 7-8; ^∗^*P* < 0.05 compared with the control group, ^∗∗^*P* < 0.01 compared with the control group.

**Figure 3:**
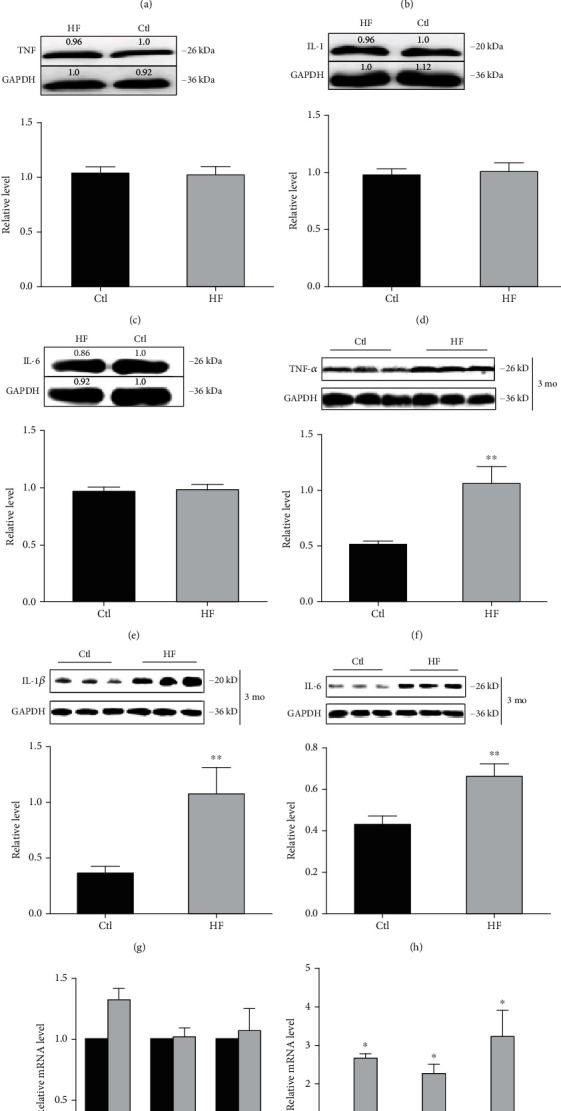
miR-181b expression was downregulated in the early stage of HF. (a, b) Real-time PCR analysis of miR-181b expression in rat heart tissue administered by ISO or saline vehicle for one month or three months, respectively. (c–e) Western blot analysis of TNF-*α*, IL-1, and IL-6 in rat heart tissue administered by ISO for one month. (f–h) Western blot analysis of TNF-*α*, IL-1*β*, and IL-6 in rat heart tissue administered by ISO for three months. (i, j) qPCR analysis of TNF-*α*, IL-1*β*, and IL-6 mRNA levels in rat heart tissue. Rat heart tissue was administered as indicated in (a) and (b). Data are expressed as mean ± SEM, *n* = 3; ^∗^*P* < 0.05 compared with the control group, ^∗∗^*P* < 0.01 compared with the control group, ^∗∗∗^*P* < 0.001 compared with the control group.

To explore the role of miR-181b in inflammation in HF, we investigated whether miR-181b regulates inflammation in HF in vivo. The HF rat model was established by intraperitoneal injection of ISO (1 mg/kg body weight) daily for one month and three months, which induced the early stage of HF and advanced HF, respectively. After one month of continuous intraperitoneal injection, each rat was examined by ultrasonic cardiogram. Though diluted heart could not be observed [17], notable cardiac hypertrophy, which is the early manifestation of HF, emerged. Compared with the control group, the group with ISO treatment for one month showed an increase in the ventricular wall (Figures 2(a) and 2(b)). In this stage of HF, the expressions of TNF-*α*, IL-1, and IL-6 in the rat heart tissue had no significant changes in contrast with the control group (*P* > 0.05; Figures 3(c)–3(e) and 3(i)). Specifically, the expression of miR-181b was downregulated by 43% statistically (*P* < 0.01; Figure 3(a)).

We further investigated the rat with ISO treatment for three months. Compared with rats injected with saline vehicle, the rats injected with ISO had dilated heart chambers (Figures 2(a) and 2(c)), decreased left ventricular ejection fraction (EF) (*P* < 0.05; Figure 2(d)), and increased left ventricular end diastolic diameter (LVEDD) (*P* < 0.01; Figure 2(e)). Interestingly, in comparison with the control, the quantitative real-time PCR (qPCR) results showed that the expression of miR-181b in the HF group was downregulated by about 56% (*P* < 0.001; Figure 3(b)), while the expression of TNF-*α*, IL-1*β*, and IL-6 was significantly increased (*P* < 0.05; Figure 3(j)). And the western blot showed similar results (*P* < 0.01; Figures 3(f)–3(h)). These findings collectively emphasize the interference role of miR-181b in inflammation during HF. Furthermore, the variation in the expression of miR-181b before the upregulation of proinflammatory cytokines in the process of HF indicates its usefulness as a biomarker for the early diagnosis of HF.

### 3.4. miR-181b Inhibits LPS-Induced Expression of Proinflammatory Cytokines In Vitro

**Figure 4:**
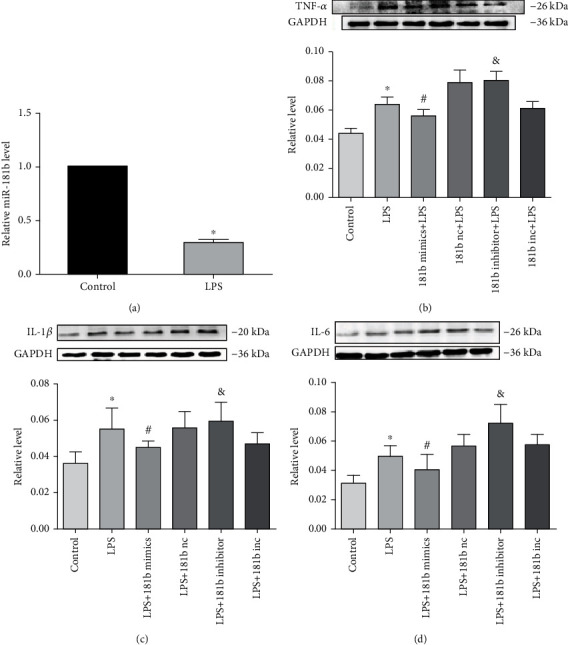
Effects of miR-181b on the expression of proinflammatory cytokines in cultured neonatal cardiomyocytes. (a) Real-time PCR analysis of miR-181b expression in cultured neonatal cardiomyocytes treated with LPS for 3 hours. (b–d) Effects of miR-181b on the expressions of TNF-*α*, IL-1*β*, and IL-6 in cultured neonatal cardiomyocytes treated with LPS. Data are expressed as mean ± SEM, *n* = 5; ^∗^*P* < 0.05 compared with the control group, ^#^*P* < 0.05 compared with the LPS+miRNA negative control group, and ^&^*P* < 0.05 compared with the LPS+miRNA inhibitor negative control.

We first analysed the expression of miR-181b in neonatal cardiomyocytes cultured with LPS for 3 hours by quantitative real-time PCR. LPS stimulates Toll-like receptor 4 (TLR4) and induces the release of crucial proinflammatory cytokines [18]. In response to the treatment, the expression of miR-181b was downregulated (*P* < 0.05; Figure 4(a)). Next, we investigated whether the overexpression of miR-181b can inhibit the inflammatory response induced by LPS under in vitro conditions. miR-181b mimics, miRNA negative control, miR-181b inhibitor, and miRNA inhibitor negative control were transfected into neonatal cardiomyocytes. 24 hours after miR-181b mimic transfection, miR-181b expressions were effectively elevated and continued for 48 hours (data not shown). Cultured neonatal cardiomyocytes were divided into six groups: control, LPS, LPS+miR-181b, LPS+miRNA negative control, LPS+ miR-181b inhibitor, and LPS+miRNA inhibitor negative control. In agreement with these results, LPS induced the expression of TNF-*α*, IL-1*β*, and IL-6 in cultured cardiomyocytes, while cotransfection with miR-181b significantly reduced this induction effect (*P* < 0.05; Figures 4(b)–4(d)). Moreover, in the presence of the miR-181b inhibitor, these protein expressions were higher than the LPS group (*P* < 0.05; Figures 4(b)–4(d)). These data indicate the inhibitory effect of miR-181b in the regulation of inflammation in cardiomyocytes.

## 4. Discussion

Our study found that the expression of miR-181b in the serum of HF patients was down-regulated, and it was negatively correlated with the level of hsCRP. We proved that miR-181b expression decreased in the early stage of HF in vivo. In support, our data strongly suggest that the inhibitory effect of miR-181b on inflammatory cytokines mediated inflammation.

During the pathological process, miRNAs are released into the blood circulation and are very stable in the plasma [19]. During HF, a variety of circulating microRNA expressions are dysregulated. Akat et al. [20] reported that circulating levels of miR-208a, miR-208b, and miR-499 were higher in advanced HF patients than healthy controls. miR-21 is the profibrotic miRNA and associated with acute myocardial infarction [21, 22]. In our study, upregulation of serum concentrations of miR-21 in ICM-induced HF patients may partly be ascribed to the above factors. Paradoxically, circulating miR-21 expressions were decreased in the DCM group, underlining a novel pivotal role in DCM that needs to be further explored.

Remarkably, we found that the circulating expression of miR-181b was reduced in patients with DCM- and ICM-induced HF and demonstrated that miR-181b had a negative correlation with hsCRP circulating levels. ROC curve analysis showed a diagnostic performance of miR-181b distinguishing DCM- and ICM-induced HF patients from healthy controls, separately. These data indicated that miR-181b might serve as a biomarker reflecting the process of HF. MicroRNAs can serve as potential biomarkers for a variety of diseases, including HF [20], while the utility of miR-181b as a biomarker for HF still needs redouble investigation.

The previous studies indicated that the miRNA-mediated effects depend on specific cell types and cellular context [23, 24]. miR-181b was identified as regulating B cell differentiation and facilitating the maturation of cytotoxic T cells [25]. Indeed, miR-181b has been detected in many cancers, including breast cancer, pancreatic and gallbladder cancer, glioblastoma multiforme, and chronic lymphocytic leukemia [25–29]. Moreover, several studies revealed that miR-181b could inhibit vascular inflammation and the initiation and progression of atherosclerosis [13, 30]. However, there are few studies on the effects of miR-181b on the progression of HF.

Inflammation participates in the progression of HF. The components of inflammatory mediators include TNF-*α* and the IL-1 family and the IL-6 family, which are the focus of the current research [3]. Previous studies have confirmed that these proinflammatory cytokines can be expressed by all nucleated cell types residing in the myocardium, including cardiomyocyte [31]. The overexpression of cytokine cascades contributes to HF progression [3]. As outlined above, modulation of proinflammatory cytokines may contribute to the improvement of HF.

We induced early stage of HF and advanced HF by intraperitoneal injection of ISO daily in a rat for a period of 1 month and 3 months. miR-181b expression was decreased by 43% when notable cardiac hypertrophy emerged but no signs of cardiac dilution. During this period, the expression of proinflammatory cytokines did not increase either. Nonetheless, after administration of ISO for three months, the expression of miR-181b was downregulated by 56%, while the expressions of TNF-*α*, IL-1*β*, and IL-6 were increased in the rat heart tissue. These data suggest its usefulness as a biomarker in the early diagnosis of HF. Moreover, it also highlights the potential correlation between miR-181b and proinflammatory cytokines (TNF-*α*, IL-1, and IL-6) in HF.

In vitro, the expression of miR-181b was downregulated in cultured neonatal cardiomyocytes treated with LPS for 3 hours, while overexpression of miR-181b inhibited LPS-induced inflammatory response. The expression of proinflammatory cytokines such as TNF-*α*, IL-1, and IL-6 increased in the LPS group and the miR-181b inhibitor group, while the miR-181b mimic group decreased. It further illustrates that miR-181b regulates inflammation in cardiomyocytes. In inflammation conditions, miR-181b may exert a protective effect on cardiomyocytes. In addition, miR-181b has been reported to regulate vascular inflammation by directly targeting importin-*α*3 and inhibiting its downstream NF-*κ*B signaling pathway in vitro and in vivo. Importin-*α*3 is a protein necessary for the nuclear transport of NF-*κ*B [32]. Yet, the mechanisms underlying miR-181b with inflammation in HF have not been fully elucidated. In the current study, we utilised gene prediction website (TargetScan, PicTar, and miRanda) and found that TNF-*α*, IL-1, and IL-6 were potential targets of miR-181b. The abovementioned signaling pathway may contribute to the further investigation of the miR-181b-related signaling pathway in the inflammation of a failing heart.

However, there are some limitations to the study that need to be noted. First, although the present study demonstrated that the miR-181b expressions were downregulated in the early stage of HF in vivo, and miR-181b inhibited proinflammatory cytokines in vitro, it is still unknown whether overexpression of miR-181b in vivo can achieve the same effect. Second, the saliva is becoming increasingly popular as a potential diagnostic tool due to its advantages such as being noninvasive, painless, and cost efficient [33]. The miR-181b expressions in the saliva of patients with HF can be detected in further studies to verify its correlation with inflammation and to find a new noninvasive method for the diagnosis of HF.

## 5. Conclusion

In summary, our research proves that the miR-181b expressions were downregulated in the early stage of HF, and it was also negatively correlated with hsCRP levels in the serum of HF patients, indicating that miR-181b may serve as a circulating biomarker for HF. Besides, our study demonstrates that miR-181b inhibited proinflammatory cytokine expressions in vitro, suggesting that miR-181b may be a new therapeutic target for the treatment of inflammation in HF. And strategies aimed at restoring miR-181b expression may provide a novel therapeutic approach to limiting inflammation in HF which may contribute to improving the prognosis of HF.

## Figures and Tables

**Table 1:** Patients' clinical data.

Clinical characteristics		ctl (n=31)	DCM (n=41)	ICM (n=31)
Ages (years)				
	Mean	50.29 ± 7.62	49.31± 8.99	55.26± 7.47
	Range	38±65	29±62	29±66
	Median	49	50	49
Sex				
	Men	14(45.2%)	27(65.9%)	21(67.7%)
	Women	17	14(34.1%)	10(32.3%)
Active smoker		7(22.5%)	14(34.1%)	16(51.6%)
History of drinking		4(12.9%)	13(31.7%)	4(12.9%)
BUN,mmol/L		4.96± 0.17	7.31± 0.45	7.35± 0.63
Creatinine,umol/L		64.75 ±2.49	86.90±7.54	98.86±15.99
UA,umol/L		299.12±18.52	455.65±22.87^∗^	373.95±24.68^∗^
ALT,u/L		22.27±2.25	40.09±6.35^∗^	81.78±44.95
AST,u/L		21.06±1.70	35.13±3.21	121.68±67.65^∗^^†^
NYHA class				
	III		21(51.2%)	16(51.61%)
	IV		20(48.8%)	15(48.4%)
Diagnosis-related factors				
	hsCRP,mg/L	2.50±0.53	6.08±1.09^∗^	43.7±10.77^∗^^†^
	NT-pro-BNP,pg/ml	60.43±6.91	3649.45±909.85^∗^	7603.3±3482.96^∗^
	LVEF,%	64.40±0.99	30.45±1.13^∗^	33.39±1.19^∗^
	LVEDD,mm	47.28±0.68	69.03±1.11^∗^	64.48±1.12^∗^
Concurrent medication,%				
	Nitroprusside sodium		65.8	83.9
	Beta-blockers		21.9	77.4
	ACEI/ARB		60.9	87
	Diuretics		82.9	96.7
	Digitoxin		63.4	67.7
	Aspirin		43.9	51.5

LVEF, left ventricular ejection fraction; LVEDD, left ventricular end diastolic diameter; NYHA class, New York Heart Association class; ACEI, angiotensin-converting enzyme inhibitor; ARB, angiotensin receptor blockers; hsCRP, high-sensitivity C-reactive protein. Data are expressed as mean±SEM or number(%); ∗*P* < 0.05 compared with control group; †*P*<0.05 compared with DCM group.

**Table 2:** All primers used in this study.

IL-1*β*
F: 5′-TGACCCATGTGAGCTGAAAG-3′
R: 5′-AGGGATTTTGTCGTTGCTTG-3′
IL-6
F: 5′-CAGGAACGAAAGTCAACTCCA-3′
R: 5′-ATCAGTCCCAAGAAGGCAACT-3′
TNF-*α*
F: 5′-GCCCACGTCGTAGCAAAC-3′'
R: 5′-GCAGCCTTGTCCCTTGAA-3′
miRNA-21
RT primer: GTCGTATCCAGTGCGTGTCGTGGAGTCGGCAATTGCACTGGATACGACTCAACAT
F: 5′-GGGAGCTTATCAGACTGATG-3′
R: 5′-TGCGTGTCGTGGAGTC-3′
miR-155
RT primer: GTCGTATCCAGTGCGTGTCGTGGAGTCGGCAATTGCACTGGATACGACACCCCTA
F: 5′-GGTGCTAATCGTGATAGGG-3′
R: 5′-AGTGCGTGTCGTGGAGTCG-3′
miR-181b
RT primer: GTCGTATCCAGTGCAGGGTCCGAGGTGCACTGGATACGACACCCACC
F: 5′-TGCGGAACATTCATTGCTGTC-3′
R: 5′-CCAGTGCAGGGTCCGAGGT-3′
U6
RT primer: CGCTTCACGAATTTGCGTGTCAT
F: 5′-GCTTCGGCAGCACATATACTAAAAT-3′
R:5′-CGCTTCACGAATTTGCGTGTCAT-3′
GAPDH
F: 5′-GCTTCGGCAGCACATATACTAAAAT -3′
R: 5′-CGCTTCACGAATTTGCGTGTCAT-3′

## Data Availability

The data used to support the findings of this study are available from the corresponding author upon request.

## References

[1] Kelkar A. A., Butler J., Schelbert E. B. (2015). Mechanisms contributing to the progression of ischemic and nonischemic dilated cardiomyopathy: possible modulating effects of paracrine activities of stem cells. *Journal of the American College of Cardiology*.

[2] Bryant D., Becker L., Richardson J. (1998). Cardiac failure in transgenic mice with myocardial expression of tumor necrosis factor-*α*. *Circulation*.

[3] Dick S. A., Epelman S. (2016). Chronic heart failure and inflammation: what do we really know?. *Circulation Research*.

[4] Van Linthout S., Tschöpe C. (2017). Inflammation-cause or consequence of heart failure or both?. *Current Heart Failure Reports*.

[5] Mingels A. M. A., Kimenai D. M. (2018). Sex-related aspects of biomarkers in cardiac disease. *Advances in Experimental Medicine and Biology*.

[6] Lee J. E. (2018). How should biobanks collect biosamples for clinical application? A 20-year biomarker-related publication and patent trend analysis. *Osong Public Health and Research Perspectives*.

[7] Malamud D. (2011). Saliva as a diagnostic fluid. *Dental Clinics of North America*.

[8] Isola G., Polizzi A., Alibrandi A., Williams R. C., Leonardi R. (2021). Independent impact of periodontitis and cardiovascular disease on elevated soluble urokinase-type plasminogen activator receptor (suPAR) levels. *Journal of Periodontology*.

[9] Isola G., Polizzi A., Alibrandi A., Williams R. C., Lo Giudice A. (2021). Analysis of galectin-3 levels as a source of coronary heart disease risk during periodontitis. *Journal of Periodontal Research*.

[10] D'Alessandra Y., Devanna P., Limana F. (2010). Circulating microRNAs are new and sensitive biomarkers of myocardial infarction. *European Heart Journal*.

[11] Hammouz R. Y., Kołat D., Kałuzińska Ż., Płuciennik E., Bednarek A. K. (2021). MicroRNAs: their role in metastasis, angiogenesis, and the potential for biomarker utility in bladder carcinomas. *Cancers*.

[12] Mir R., Elfaki I., Khullar N. (2021). Role of selected miRNAs as diagnostic and prognostic biomarkers in cardiovascular diseases, including coronary artery disease, myocardial infarction and atherosclerosis. *Journal of Cardiovascular Development and Disease*.

[13] Sun P., Li L., Liu Y. Z. (2019). MiR-181b regulates atherosclerotic inflammation and vascular endothelial function through Notch 1 signaling pathway. *European Review for Medical and Pharmacological Sciences*.

[14] Ponikowski P., Voors A. A., Anker S. D. (2016). 2016 ESC guidelines for the diagnosis and treatment of acute and chronic heart failure: the task force for the diagnosis and treatment of acute and chronic heart failure of the European society of cardiology (ESC) developed with the special contribution of the heart failure association (HFA) of the ESC. *European Heart Journal*.

[15] Pinto Y. M., Elliott P. M., Arbustini E. (2016). Proposal for a revised definition of dilated cardiomyopathy, hypokinetic non-dilated cardiomyopathy, and its implications for clinical practice: a position statement of the ESC working group on myocardial and pericardial diseases. *European Heart Journal*.

[16] Felker G. M., Shaw L. K., O'Connor C. M. (2002). A standardized definition of ischemic cardiomyopathy for use in clinical research. *Journal of the American College of Cardiology*.

[17] Oyamada M., Kimura H., Oyamada Y., Miyamoto A., Ohshika H., Mori M. (1994). The expression, phosphorylation, and localization of connexin 43 and gap- junctional intercellular communication during the establishment of a synchronized contraction of cultured neonatal rat cardiac myocytes. *Experimental Cell Research*.

[18] Lu Y. C., Yeh W. C., Ohashi P. S. (2008). LPS/TLR4 signal transduction pathway. *Cytokine*.

[19] Chen X., Ba Y., Ma L. (2008). Characterization of microRNAs in serum: a novel class of biomarkers for diagnosis of cancer and other diseases. *Cell Research*.

[20] Akat K. M., Moore-McGriff D., Morozov P. (2014). Comparative RNA-sequencing analysis of myocardial and circulating small RNAs in human heart failure and their utility as biomarkers. *Proceedings of the National Academy of Sciences*.

[21] Guo H., Zhao X., Su H. (2020). miR-21 is upregulated, promoting fibrosis and blocking G2/M in irradiated rat cardiac fibroblasts. *PeerJ*.

[22] Chen C. H., Hsu S. Y., Chiu C. C., Leu S. (2019). MicroRNA-21 mediates the protective effect of cardiomyocyte-derived conditioned medium on ameliorating myocardial infarction in rats. *Cell*.

[23] Cheng A. M., Byrom M. W., Shelton J., Ford L. P. (2005). Antisense inhibition of human miRNAs and indications for an involvement of miRNA in cell growth and apoptosis. *Nucleic Acids Research*.

[24] Liu X., Cheng Y., Yang J., Xu L., Zhang C. (2012). Cell-specific effects of miR-221/222 in vessels: molecular mechanism and therapeutic application. *Journal of Molecular and Cellular Cardiology*.

[25] Di Marco M., Veschi S., Lanuti P. (2021). Enhanced expression of miR-181b in B cells of CLL improves the anti-tumor cytotoxic T cell response. *Cancers*.

[26] Taha M., Mitwally N., Soliman A. S., Yousef E. (2020). Potential diagnostic and prognostic utility of miR-141, miR-181b1, and miR-23b in breast cancer. *International Journal of Molecular Sciences*.

[27] Liu G., Shao C., Li A., Zhang X., Guo X., Li J. (2020). Diagnostic value of plasma miR-181b, miR-196a, and miR-210 combination in pancreatic cancer. *Gastroenterology Research and Practice*.

[28] Wu K., Huang J., Xu T. (2019). MicroRNA-181b blocks gensenoside Rg3-mediated tumor suppression of gallbladder carcinoma by promoting autophagy flux via CREBRF/CREB3 pathway. *American Journal of Translational Research*.

[29] Yin J., Shi Z., Wei W. (2020). miR-181b suppress glioblastoma multiforme growth through inhibition of SP1-mediated glucose metabolism. *Cancer Cell International*.

[30] Di Gregoli K., Mohamad Anuar N. N., Bianco R. (2017). MicroRNA-181b controls atherosclerosis and aneurysms through regulation of TIMP-3 and elastin. *Circulation Research*.

[31] Saraf A., Rampoldi A., Chao M. (2021). Functional and molecular effects of TNF-*α* on human iPSC-derived cardiomyocytes. *Stem Cell Research*.

[32] Sun X., Icli B., Wara A. K. (2012). MicroRNA-181b regulates NF-*κ*B-mediated vascular inflammation. *The Journal of Clinical Investigation*.

[33] Sapkota D., Søland T. M., Galtung H. K. (2020). COVID-19 salivary signature: diagnostic and research opportunities. *Journal of Clinical Pathology*.

